# Identification of a Multi–Long Noncoding RNA Signature for the Diagnosis of Type 1 Diabetes Mellitus

**DOI:** 10.3389/fbioe.2020.00553

**Published:** 2020-07-03

**Authors:** Guannan Geng, Zicheng Zhang, Liang Cheng

**Affiliations:** ^1^Department of Endocrinology, The First Affiliated Hospital of Harbin Medical University, Harbin, China; ^2^College of Bioinformatics Science and Technology, Harbin Medical University, Harbin, China

**Keywords:** long noncoding RNAs, biomarkers, type 1 diabetes mellitus, diagnosis, signature

## Abstract

Due to the increasing prevalence of type 1 diabetes mellitus (T1DM) and its complications, there is an urgent need to identify novel methods for predicting the occurrence and understanding the pathogenetic mechanisms of the disease. Accumulated data have demonstrated the potential of long noncoding RNAs (lncRNAs), as biomarkers in establishing diagnosis and predicting prognosis of numerous diseases. Yet, little is known about the expression patterns and regulatory roles of lncRNAs in the pathogenesis of T1DM and whether they can be used as diagnostic biomarkers for the disease. To further explore these questions, in the present study, we conducted a comparative analysis of the expression patterns of lncRNAs between 20 T1DM patients and 42 health controls by retrospectively analyzing a published microarray data set. Our results indicate that, compared with healthy controls, diabetic patients had altered levels of lncRNAs. Then, we used three time cross-validation strategy and support vector machine to propose a specific 26-lncRNA signature (termed 26LncSigT1DM). This 26LncSigT1DM signature can be used to effectively distinguish between healthy and diabetic individuals (area under the curve = 0.825) of a validation cohort. After the 26LncSigT1DM was prospectively validated, we used Pearson correlation to identify 915 mRNAs, whose expression levels were positively correlated with those of the 26 lncRNAs. According to their Gene Ontology annotations, these mRNAs participate in processes including cellular response to stimulus, cell communication, multicellular organismal process, and cell motility. Kyoto Encyclopedia of Genes and Genomes analysis demonstrated that the genes encoding the 915 mRNAs may be associated with the NOD-like receptor signaling pathway, transforming growth factor β signaling pathway, and mineral absorption, suggesting that the deregulation of these lncRNAs may mediate inflammatory abnormalities and immune dysfunctions, which jointly promote the pathogenesis of T1DM. Thus, our study identifies a novel diagnostic tool and may shed more light on the molecular mechanisms underlying the pathogenesis of T1DM.

## Introduction

As one of the most notorious autoimmune disorders, type 1 diabetes mellitus (T1DM) is a chronic childhood-onset disease caused by selective destruction of pancreatic islet beta cells (Petersmann et al., 2018). Pathogenic factors of T1DM include epigenetic, environmental, and genetic factors (Bluestone et al., 2010; Groop and Pociot, 2014). Additionally, it has been demonstrated that both immune dysfunctions and islet beta cell defects contribute to the pathogenesis of T1DM (Lennon et al., 2009; Bluestone et al., 2010). During the onset of the disease β-cell function in T1DM patients may have already been completely destroyed (Polychronakos and Li, 2011). Therefore, it is necessary to diagnose T1DM early in its development. However, as onsets of T2DM and other types of diabetes occurring earlier, traditional methods for diagnosing diabetes are no longer satisfactory (Zou et al., 2018). Therefore, there is an urgent need to understand the causal factors and pathogenetic mechanisms of T1DM for more effective diagnosis and treatment.

Long noncoding RNAs (lncRNAs) are a group of RNAs that are longer than 200 nucleotides and do not encode proteins (Ponting et al., 2009; Sun et al., 2013; Zhang et al., 2017; Su et al., 2018; Yao et al., 2018). Recent advancement in large-scale genomic analysis has greatly enriched our knowledge on this type of RNA. Increasing evidence suggests that lncRNAs are involved in a wide range of cellular biological processes (Huang et al., 2012; Zhang et al., 2017; Guo et al., 2018), including cell proliferation (Jin et al., 2017; Luo et al., 2017), differentiation (Alvarez-Dominguez et al., 2015), and apoptosis (Lu et al., 2016). Specifically, βlinc1 (formerly referred to as HI-LNC15), a lncRNA uniquely expressed in the islet, has been reported to facilitate the proper specification and maintain the normal function of islet β cells (Arnes et al., 2016). Interestingly, in βlinc1-deficient mature islet β cells, the expression of GLIS3, a causative gene for both T1DM and T2DM, was downregulated (Moran et al., 2012). MEG3 is another lncRNA implicated in diabetes. In mouse islet β cells, MEG3 has been demonstrated to play important roles in insulin synthesis and secretion (You et al., 2016).

Recently, researchers have shown that lncRNAs control the differentiation and function of innate and adaptive immune cells to coordinate different immune functions (Atianand and Fitzgerald, 2014; Chen et al., 2017). In a previous study, it was confirmed that lincRNA LincR-Ccr2-5′AS plays an important role in regulating the expression of T_H_2-specific genes and is essential for migration of T_H_2 cells (Hu et al., 2013). Furthermore, LincRNACox2 and lncRNA THRIL are two lncRNAs that are crucial for inflammatory activation because they can regulate the TLR signaling pathways (Carpenter et al., 2013). Taken together, all the aforementioned studies suggest that lncRNAs can regulate both the activation of the innate immune system and islet β cell function, the defects of which contribute significantly to the pathogenesis of T1DM. More importantly, accumulating evidence has shown that dysregulated lncRNA expression is associated with the development of T1DM (Motterle et al., 2015; Mirza et al., 2017), suggesting that lncRNAs could be used as biomarkers to assess the risk of T1DM. However, neither the expression patterns of lncRNAs in T1DM patients nor their potential as T1DM biomarkers has been thoroughly investigated.

To provide more insights into the expression patterns of lncRNAs in T1DM patients and evaluate their potential as T1DM biomarkers, in this study, we comparatively analyzed lncRNA expression levels in 42 healthy individuals and 20 T1DM patients based on a published microarray data set and identified a group of differentially expressed lncRNAs. We also demonstrated that these lncRNAs may represent a multi–long noncoding RNA signature (namely 26LncSigT1DM) that can be used to effectively distinguish between healthy and diabetic individuals and identify T1DM susceptible individuals. After the 26LncSigT1DM signature was prospectively validated, we identified 915 mRNAs whose expression levels are positively correlated with those of the 26LncSigT1DM lncRNAs. Functional analysis of these mRNAs indicates that they are involved in a variety of biological processes, including cellular function and communication, and that the genes encoding these mRNAs are associated with pathways that can potentially mediate inflammatory abnormalities and immune dysfunctions. Our study provides a platform for developing 26LncSigT1DM into a diagnostic tool for T1DM and for future research into the molecular mechanisms underlying the pathogenesis of T1DM.

## Materials and Methods

### Participant Information

We included two cohorts of individuals in this study. One cohort (62 individuals, accession number GSE35713) was from Hara's study (Levy et al., 2012), and the data are from peripheral blood mononuclear cells in the plasma samples of patients with new-onset T1DM. The other (22 individuals, GSE55100) was documented in Yang et al. (2015). Patients without survival time or events were excluded.

### Data Acquisition and lncRNA Expression Analysis

We obtained the raw microarray data (.CEL format) deposited in the Gene Expression Omnibus database from the individuals mentioned above. To ensure uniformity, we used the Robust Multichip Average algorithm to preprocess the data (Irizarry et al., 2003). To scale probe expression intensity, the data set was quantile-normalized and log2-transformed after background correction, and then it was independently standardized by *Z* score transformation (Cheadle et al., 2003).

Gene expression profiles of the individuals had been previously analyzed by an Affymetrix Human Genome U133 Plus 2.0 array (HG-U133 Plus_2.0). We visited the Affymetrix website (http://www.affymetrix.com) to obtain the probe sequences used in the array.

By repurposing the Affymetrix array probes, lncRNA expression profiles of the 62 individuals in cohort GSE35713 were determined as described in previous studies (Du et al., 2013; Zhou et al., 2015). Briefly, we mapped the probes to the human genome (GRCh38) using the SeqMap tool (Jiang and Wong, 2008) and used GENCODE Release 21 to determine lncRNA-encoding genes. If a probe corresponds to numerous lncRNAs, it will be directly abandoned. If an lncRNA is targeted by multiple probes, its expression value was defined as the average value of the expression levels determined by all the corresponding probes. Using this method, we were able to obtain the expression profiles of 1,326 lncRNAs.

To identify differentially expressed lncRNAs (DELs), we compared the lncRNA expression patterns between healthy and T1DM individuals using two-tailed *t*-tests. Bonferroni statistical tests were then carried out, and lncRNAs with a Bonferroni-corrected *p*-value below 0.01 (Supplementary Table 1) were considered as DELs, whereas those with a Bonferroni-corrected *p* < 0.05 but higher than 0.01 (Supplementary Table 2) were finally abandoned. To assess the potential of the DELs as biomarkers for T1DM, unsupervised hierarchical clustering analysis was carried out using R package based on the Euclidean distance and the complete linkage method.

### Identification of lncRNAs Associated With T1DM

To propose a diagnostic lncRNA molecular signature for T1DM, we used a sigmoid kernel-based support vector machine (SVM) (Tang et al., 2018; Lai et al., 2019) and assessed the predictive ability of the model using 3-fold cross-validation, with 62 individuals in cohort GSE35713 being defined as the discovery cohort. The details are as follows:

- Individuals in the discovery cohort were equally divided into three nonoverlapping sets.
- Candidate lncRNAs were sorted according to their importance in the random forest classification algorithm. Then, a supervised discriminative model was established, and lncRNAs with a Bonferroni-corrected *p* < 0.01 (Supplementary Table 1) were selected.
- Distinguishing T1DM patients from healthy controls using the SVM-based signature based on voting rules: We added one candidate lncRNA each time sequentially according to the rankings of candidate lncRNAs in the list (i.e., the lncRNA ranked first in the list was added first). After the three nonoverlapping sets were applied, the performance of the SVM-based signature was evaluated.
- The optimal number of lncRNAs in the SVM-based signature could be determined after the balance between lncRNA number and discrimination accuracy was achieved.

### Performance Evaluation

The difference between healthy and T1DM individuals was plotted (with SVM) (Su et al., 2018; Yang et al., 2018; Zhu et al., 2019), of which the performance was tested by three nonoverlapping sets. A 2 × 2 contingency table was used to calculate the sensitivity, specificity, and accuracy of the area under the curve (AUC). We plotted true-positive rates (sensitivity) against false-positive rates (1—specificity) to generate the receiver operating characteristic (ROC) curve (Lv et al., 2019), which was then used to determine the discrimination efficiency. We employed Bioconductor and R package to conduct all the aforementioned analyses.

### Functional Annotations of the 26 lncRNAs in the 26LncSigT1DM Signature

**Figure 1:**
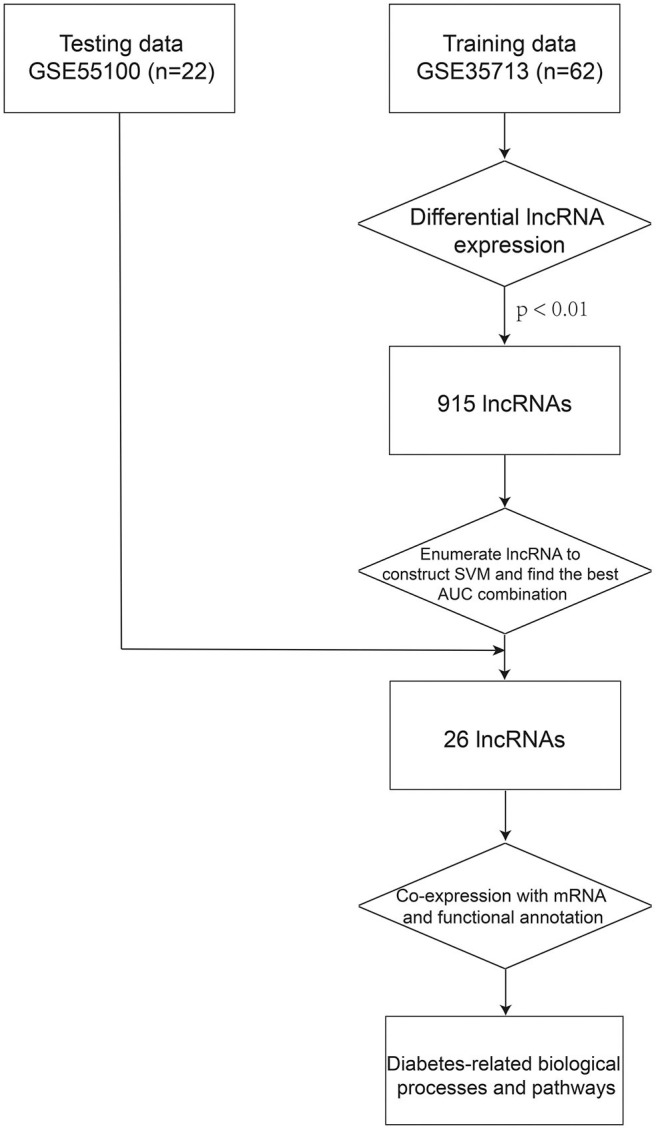
The process of the method.

To predict biological functions of the 26 lncRNAs in the 26LncSigT1DM signature, the Pearson correlation coefficient was adopted to determine correlations between expression levels of mRNAs and those of the 26 lncRNAs. Then, the genes encoding the mRNAs whose expression levels are positively correlated with those of the 26 lncRNAs were subjected to the Gene Ontology (GO) and Kyoto Encyclopedia of Genes and Genomes (KEGG) functional annotations using the DAVID (Database for Annotation, Visualization, and Integrated Discovery, version 6.7) (Huang da et al., 2009). Biological processes enriched in the GO analysis with a Benjamini–Hochberg–adjusted *p* < 0.01 and an enrichment score >1.5 were considered significant. Similarly, pathways enriched in the KEGG analysis with a Benjamini–Hochberg–adjusted *p* < 0.01 and an enrichment score >1.5 were also considered significant (Figure 1).

## Results

### Identification of DELs Between Healthy and Diabetic Individuals

Hara's cohort (GSE35713), the larger cohort included in our study, contained 47 RO T1DM patients and 42 unrelated healthy controls (Levy et al., 2012). To identify RO T1DM-related lncRNAs, we selected 20 T1DM patients and 42 unrelated healthy controls and defined them as a discovery cohort (*n* = 62). Then, we compared lncRNA expression levels between the 20 T1DM patients and 42 healthy controls by performing SAM analysis. After abandoning lncRNAs with a false discovery rate (FDR)–adjusted *p* < 0.01, we totally identified 1,326 DELs (log FC > 1 or log FC ≤ 1, FDR-adjusted *p* < 0.01, Supplementary Table 3).

### Construction of SVM-Based and Multi-lncRNA Signature as a Diagnostic Tool for T1DM Using the Discovery Cohort

**Figure 2:**
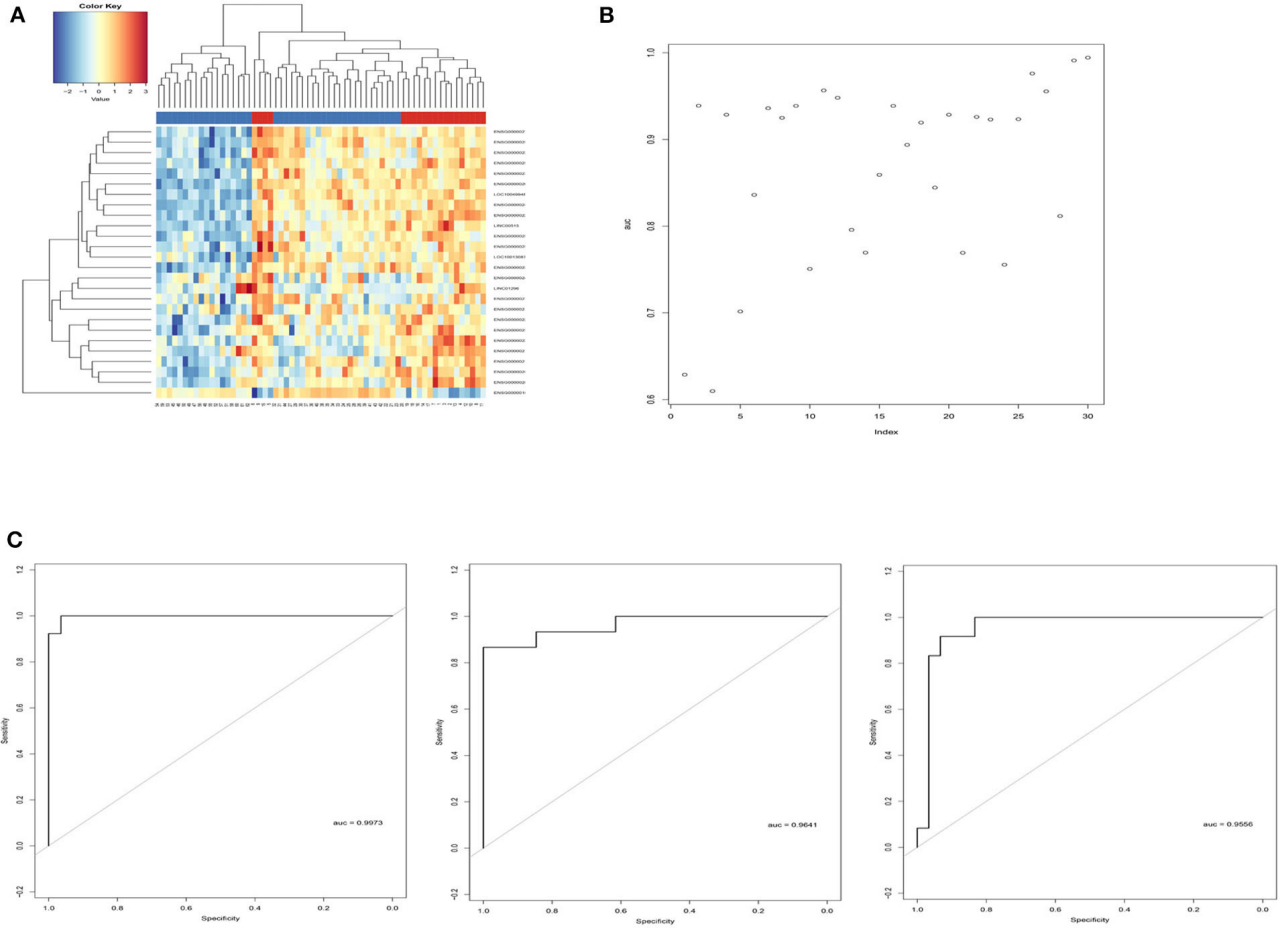
Identification of the SVM and 3-fold cross-validation–based multi-lncRNA signature and its application in T1DM diagnosis. **(A)** Hierarchical clustering analysis of the 62 individuals in the discovery cohort based on the expression levels of the 26 lncRNAs in the 26LncSigT1DM signature. **(B)** Performance of different lncRNA numbers in distinguishing healthy and diabetic individuals of the discovery cohort. **(C)** Performance of the SVM and 3-fold cross-validation–based 26LncSigT1DM signature in distinguishing healthy and diabetic individuals of the discovery cohort.

Sigmoid kernel-based SVM and 3-fold cross-validation strategies were used to search for a supervised T1DM predictor from the discovery cohort. We identified a signature of 26 lncRNAs, which were downregulated in T1DM patients in Hara's cohort, had the highest discrimination accuracy (Figures 2A,B). This signature was named 26LncSigT1DM (Table 1). At the same time, we found that 26 lncRNAs is also an optimal number of lncRNAs that balances lncRNA number and discrimination accuracy. By distinguishing healthy and diabetic individuals in Hara's cohort with this 26LncSigT1DM signature, we generated three ROC curves, whose AUCs are 0.9973, 0.9641, and 0.9556 (Figure 2C). Hierarchical clustering was then applied to analyze the expression profiles of the 26 lncRNAs in the 26lncSigT1DM signature in the healthy and diabetic individuals. We found that the 20 T1DM patients and the 42 healthy controls can be grouped into two significantly different clusters (the 20 T1DM patients were grouped into Cluster 1, whereas the 42 healthy controls were grouped into Cluster 2) based on the expression levels of the 26 lncRNAs in the 26lncSigT1DM signature (*p* = 3.579e-05, χ^2^-test). Therefore, we successfully distinguished between healthy and diabetic individuals in the discovery cohort using the 26lncSigT1DM signature. These results suggest that the downregulation of the 26 lncRNAs in the 26lncSigT1DM signature is able to reflect the disease progression of T1DM and that the signature has a great potential to be used as a diagnostic tool for T1DM.

**Table 1:** Detailed information of the 26 lncRNAs in the 26LncSigT1DM signature.

**Gene name**	**Gene. Name**	**Score**	**Numerator**	**Denominator**	**Fold change**	***Q*-value**
ENSG00000224020	MIR181A2HG	6.193	0.57	0.092	1.103	0
ENSG00000253165	–	2.504	0.185	0.074	1.066	0
ENSG00000259150	LINC00929	3.433	0.313	0.091	1.092	0
ENSG00000248118	LINC01019	3.363	0.305	0.091	1.104	0
ENSG00000231365	WARS2-AS1	3.151	0.19	0.06	1.06	0
LINC01296	DUXAP9	2.359	0.391	0.166	1.154	0.085
LINC00515	LINC00515	4.022	0.336	0.083	1.138	0
ENSG00000203999	LINC01270	2.826	0.25	0.088	1.088	0
ENSG00000227540	–	3.261	0.251	0.077	1.053	0
ENSG00000278156	TSC22D1-AS1	2.35	0.173	0.074	1.056	0.085
ENSG00000236519	LINC01424	2.352	0.195	0.083	1.082	0.085
ENSG00000257242	LINC01619	3.532	0.4	0.113	1.077	0
LOC100130872	–	2.785	0.253	0.091	1.049	0
ENSG00000215417	MIR17HG	4.629	0.342	0.074	1.107	0
ENSG00000186594	MIR22HG	−7.497	−0.701	0.093	0.924	0
ENSG00000275549	STPG3-AS1	2.528	0.193	0.076	1.043	0
ENSG00000214401	KANSL1-AS1	2.933	0.221	0.075	1.055	0
ENSG00000237940	LINC01238	2.842	0.269	0.095	1.094	0
ENSG00000212978	–	2.538	0.205	0.081	1.055	0
ENSG00000246339	EXTL3-AS1	2.152	0.135	0.063	1.039	0.085
ENSG00000281649	EBLN3P	3.874	0.226	0.058	1.029	0
ENSG00000223478	–	6.081	0.566	0.093	1.113	0
ENSG00000258573	–	2.564	0.204	0.079	1.048	0
LOC100499489	–	3.018	0.244	0.081	1.065	0
ENSG00000254813	–	3.51	0.336	0.096	1.082	0
ENSG00000229589	ACVR2B-AS1	3.157	0.36	0.114	1.091	0

### Validation of the 26LncSigT1DM Signature

**Figure 3:**
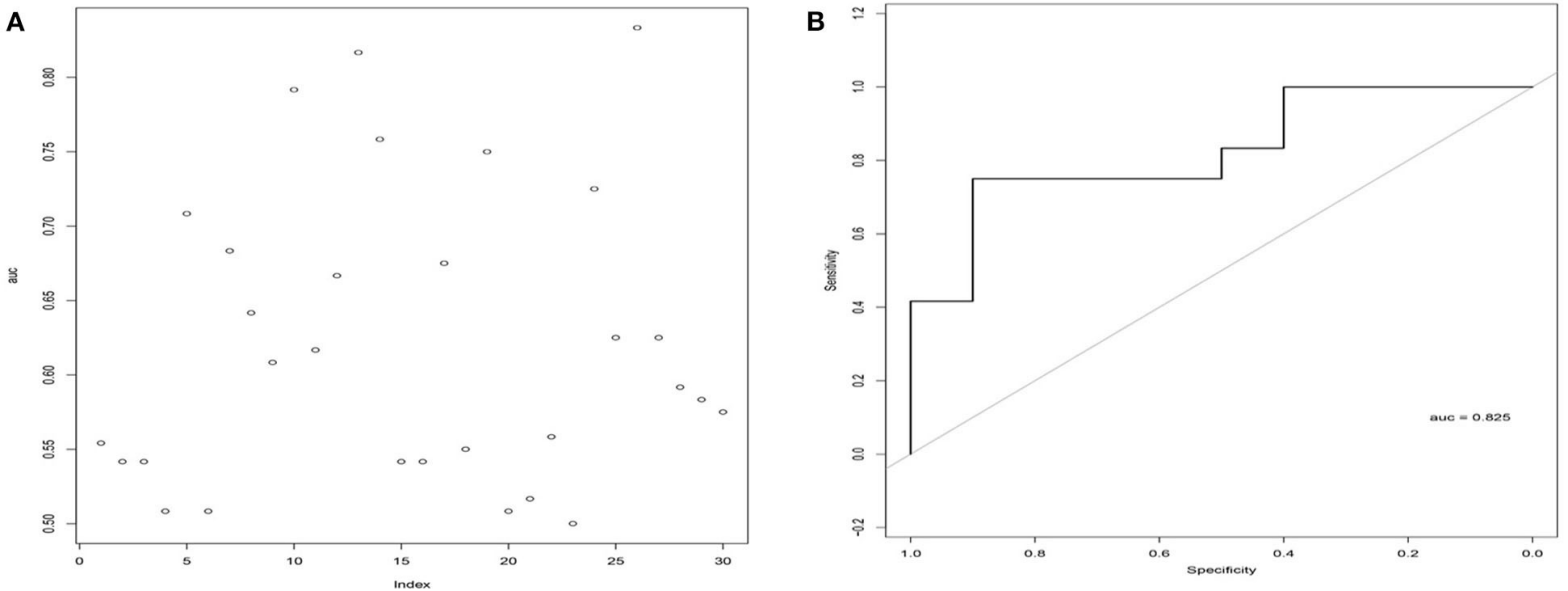
Validation of the 26LncSigT1DM signature using an additional independent cohort. **(A)** Performance of different lncRNA numbers in distinguishing healthy and diabetic individuals of the validation cohort. **(B)** Performance of the SVM and 3-fold cross-validation–based 26LncSigT1DM signature in distinguishing healthy and diabetic individuals of the validation cohort.

To test the stability and robustness of the 26LncSigT1DM, we introduced another cohort of 22 individuals (including 12 T1DM patients and 10 healthy controls) from Minglan's study (Du et al., 2013). This validation cohort was analyzed with the 26LncSigT1DM signature using sigmoid kernel-based SVM and 3-fold cross-validation strategies. According to our results, by distinguishing healthy and diabetic individuals in the validation cohort with the 26LncSigT1DM signature, we generated an ROC curve with an AUC of 0.825 (Figures 3A,B), suggesting that the proposed 26LncSigT1DM signature is a reliable diagnostic tool for T1DM.

### Exploration of the Biological Functions of the 26 lncRNAs in the 26LncSigT1DM Signature

**Figure 4:**
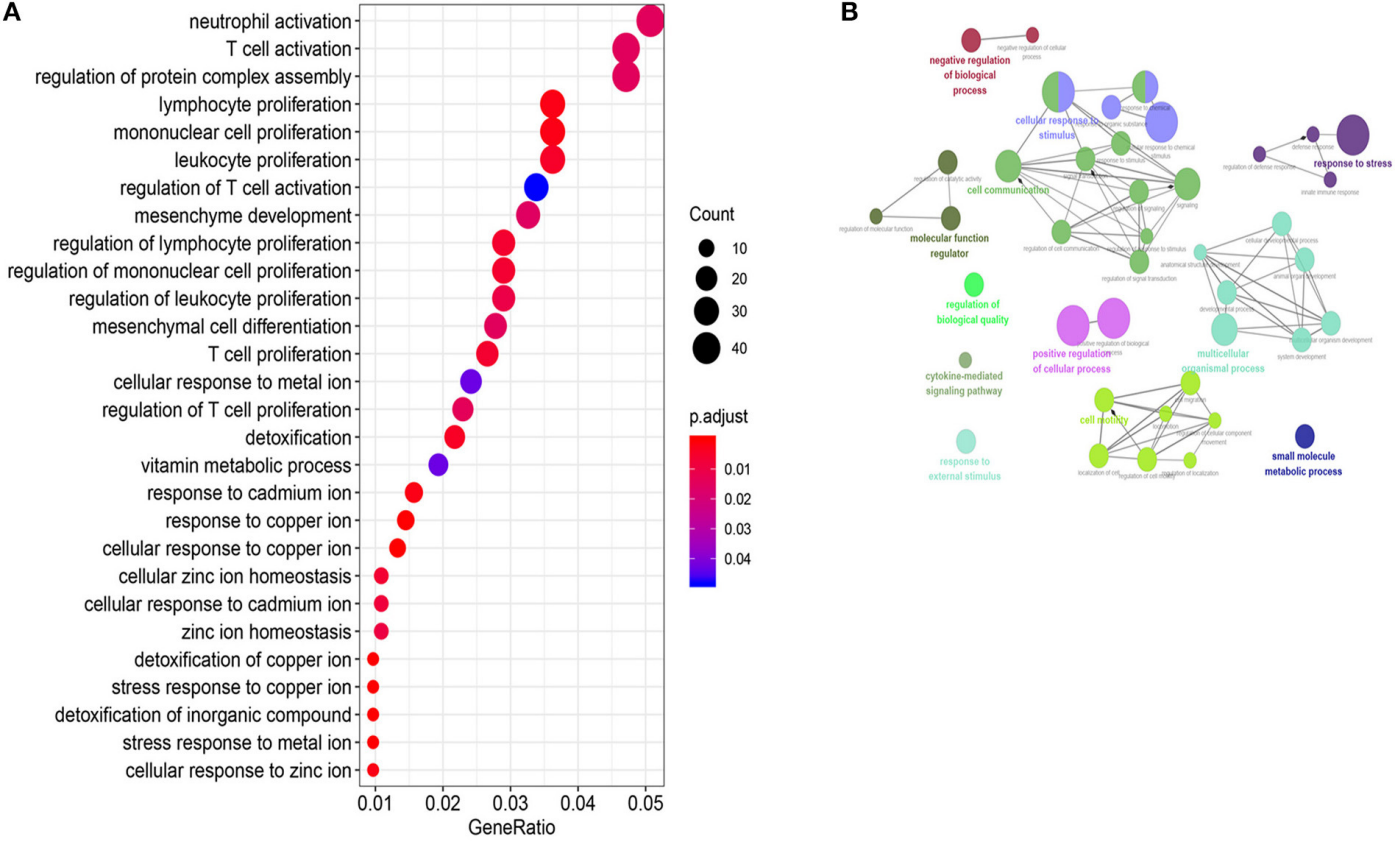
Function annotations of the 26LncSigT1DM lncRNAs. **(A)** Gene Ontology and Kyoto Encyclopedia of Genes and Genomes annotations of the genes encoding the mRNAs whose expression levels are positively correlated with those of the 26 lncRNAs in the 26LncSigT1DM signature. The results indicate that the 26LncSigT1DM lncRNAs may affect immune processes. **(B)** The lncRNAs involved in regulating human biological processes.

To predict biological functions of the 26 lncRNAs in the 26LncSigT1DM signature, we adopted the Pearson correlation coefficient to determine correlations between expression levels of mRNAs and those of the 26 lncRNAs. We found 915 mRNAs whose expression levels were positively correlated with those of the 26 lncRNAs. According to our GO analysis of the genes encoding these mRNAs, 470 biological processes were significantly enriched (Supplementary Table 4, *p*-values < 0.05, Figure 4A). These biological processes can be clustered into four major functional groups, including cellular response to stimulus, cell communication, multicellular organismal process, and cell motility (Figure 4B). KEGG analysis of the genes encoding the 915 mRNAs indicates that they are implicated in several pathways including the NOD-like receptor signaling pathway, transforming growth factor β (TGF-β) signaling pathway, autoimmune thyroid disease, and mineral absorption (Supplementary Table 5). Given that all the biological processes and signaling pathways enriched in our GO and KEGG analyses are associated with the pathogenesis of T1DM, we speculate that the downregulation of the 26 lncRNAs may have caused the aberrant expression of a wide range of genes, which subsequently contributes to the pathogenesis of T1DM.

## Discussion

Type 1 diabetes mellitus, one of the most common childhood-onset chronic diseases, is caused by defects in pancreatic islet β cells. Complications of T1DM are very serious and sometimes fatal. For example, diabetic ketoacidosis is a life-threatening complication of T1DM caused by a shortage of insulin that demands insulin injections and blood glucose monitor. Recent years have witnessed a rising incidence of diabetes, which has been correlated with both environmental conditions and genetic factors (Fourlanos et al., 2008). To cope with this growing problem, great efforts and progress have been made in the last few years to explore the possible mechanisms underlying the pathogenesis of T1DM at the miRNA, mRNA, and protein levels. So far, several miRNA/mRNA/protein-based signatures have been associated with the occurrence of T1DM, which facilitates the development of new diagnostic and prognostic tools (Azhir et al., 2018; Liao et al., 2018; Bertoccini et al., 2019; Cheng et al., 2019; Guay et al., 2019).

Long noncoding RNAs are a novel group of gene expression regulators (Gibb et al., 2011; Kung et al., 2013). Increasing evidence has revealed that lncRNAs control the differentiation and function of innate and adaptive immune cells to coordinate several aspects of immune functions (Carpenter et al., 2013; Hu et al., 2013; Atianand and Fitzgerald, 2014; Chen et al., 2017). Therefore, their associations with autoimmune diseases have become a research hotspot. To date, several studies have demonstrated the potential of lncRNAs as novel diagnostic or prognostic tool for various types of cancer (Du et al., 2013; Zhou et al., 2015, 2017; Bao et al., 2019). Although several lncRNAs, including HILNC25, lncRNA MEG3, and MALAT-1, have been found to contribute to diabetes (Arnes et al., 2016; Lu et al., 2016; You et al., 2016), little is known about the expression profiles of lncRNAs in T1DM patients and whether lncRNAs can be used as diagnostic or prognostic tool for T1DM.

In the present study, we retrospectively analyzed a published microarray data set and determined the expression levels of lncRNAs in 62 individuals (the discovery cohort), including 20 T1DM patients and 42 healthy controls. Using this discovery cohort, we identified a supervised multi-lncRNA T1DM diagnostic signature, 26LncSigT1DM, based on SVM and 3-fold cross-validation strategies. This 26LncSigT1DM signature consists of 26 lncRNAs, whose expression levels were downregulated in the 20 T1DM patients as compared with the 42 healthy controls. Using the 26LncSigT1DM signature, we accurately distinguished between healthy and diabetic individuals in the discovery cohort. To test the stability and robustness of the 26LncSigT1DM signature, we introduced a 22-individual validation cohort (a cohort from Minglan's study) and found that the 26LncSigT1DM signature was also able to accurately distinguish between healthy and diabetic individuals in the validation cohort. According to the tree traversal algorithms, the number of combined lncRNAs was found to be not correlated with the model effects. Therefore, to avoid overfitting or underfitting, the combination of 26 lncRNA models was finally selected to build the classifier. These results suggest that the proposed 26LncSigT1DM signature has a great potential to be used as a diagnostic tool for T1DM. To the best of our knowledge, this is the first multi-lncRNA signature capable of diagnosing T1DM early in its development. However, there are several limitations in this study. First, only two microarray datasets are available online, limiting the sample size analyzed in this study. Second, since the signature of T1DM was derived from newly-onset patients without the data after onset, the 26LncSigT1DM cannot be applied to the prognosis analysis. Last but not least, due to limited available data of T2DM patients, this signature is not capable of distinguishing T1DM from T2DM. Future studies related to these questions are worth conducting.

Prior studies have confirmed that lncRNAs are important gene expression regulators because they modulate the expression of a wide range of functional genes involved in multiple biological processes (Guo et al., 2013; Liu et al., 2015). To predict biological functions of the 26 lncRNAs in the 26LncSigT1DM signature, we used the Pearson correlation coefficient to identify correlations between expression levels of mRNAs and those of the 26 lncRNAs. We found 915 mRNAs whose expression levels were positively correlated with those of the 26 lncRNAs. According to our GO and KEGG analyses, the genes encoding these mRNAs are involved in multiple T1DM-related biological processes and signaling pathways. These results are consistent with previous findings. In a previous study, it was reported that thioredoxin-interacting protein (TXNIP), an activator of NOD, LRR, and PYD domains-containing protein 3 (NLRP3) inflammasome, is associated with nonalcoholic fatty liver disease and T1DM (Wang et al., 2013). In addition, it was demonstrated that mitochondrial DNA-mediated NLRP3 activation can induce IL-1β secretion in the pancreas of STZ-induced T1DM mice (Carlos et al., 2017). The TGF-β signaling pathway inside T cells, which coordinates immune responses, has already been proved to play a critical role in the pathogenesis of T1DM (Green et al., 2003). Type 1 diabetes mellitus patients have already been thought to have increased risk to suffer from autoimmune thyroid disease compared with healthy individuals. Our KEGG analysis implies that autoimmune thyroid disease and mineral absorption may be closely related to T1DM. Among these 26 lncRNAs, LINC01619 alteration has been proved to influence the diabetic nephropathy by inducing oxidative stress and podocyte damage via regulating miR-27a (Bai et al., 2018). However, except for LINC01619, the mecanisms of other lncRNAs affecting T1DM still remain unclear, which need to be clarified. The results of the GO and KEGG analyses of the 26LncSigT1DM signature in this study pave the way for further studies to investigate the relationship between these lncRNAs and T1DM as well as its complications. The underlying mechanisms remain to be further studied. Thus, the results of our study may provide suggestive information for future research.

In summary, we conducted a comparative analysis of the lncRNA expression profiles between T1DM patients and healthy controls. And a dysregulated lncRNA-mRNA coexpression network was built to enrich our knowledge of T1DM-related lncRNAs. More importantly, we proposed and validated a 26LncSigT1DM signature that has a great potential to be used as a diagnostic tool for T1DM using sigmoid kernel-based SVM and 3-fold cross-validation strategies. This study is the first to use a multi-lncRNA signature to diagnose T1DM. Therefore, the 26LncSigT1DM signature proposed by our study may represent a good complement to the existing clinical diagnostic indicators for T1DM. Lastly, this study also improves our understanding of the mechanisms underlying the pathogenesis of T1DM and may provide other options for the prevention and treatment of T1DM.

## Data Availability Statement

Publicly available datasets were analyzed in this study. This data can be found here: GSE35713, https://www.ncbi.nlm.nih.gov/geo/query/acc.cgi?acc=GSE35713, GSE55100, https://www.ncbi.nlm.nih.gov/geo/query/acc.cgi?acc=GSE55100.

## Author Contributions

GG, ZZ, and LC designed and performed the experiments, analyzed the data, wrote the manuscript, organized the figures, and revised the manuscript. LC also improved the experimental performance, revised, finalized, and approved the article. All authors contributed to the article and approved the submitted version.

## Conflict of Interest

The authors declare that the research was conducted in the absence of any commercial or financial relationships that could be construed as a potential conflict of interest.
